# Codeine as a Potential Drug Against Motor Symptoms in Parkinson’s Disease: Report of Two Cases

**DOI:** 10.7759/cureus.94943

**Published:** 2025-10-19

**Authors:** Takeo Kato, Shingo Koyama, Takeshi Hayashi, Yasuyuki Ohta, Shigeki Arawaka

**Affiliations:** 1 Department of Neurology, National Hospital Organization Yamagata National Hospital, Yamagata, JPN; 2 Department of Neurology and Cerebrovascular Medicine, Saitama Medical University International Medical Center, Hidaka, JPN; 3 Division of Neurology, Department of Internal Medicine III, Yamagata University Faculty of Medicine, Yamagata, JPN; 4 Division of Neurology, Department of Internal Medicine IV, Osaka Medical and Pharmaceutical University Faculty of Medicine, Osaka, JPN

**Keywords:** akinesia, bradykinesia, codeine phosphate, drug-repositioning, pain, parkinsonism, wearing-off

## Abstract

Although many drugs for Parkinson’s disease (PD) with different modes of action are employed in clinical settings, the wearing-off phenomenon occurs frequently in the advanced stages of the disease; thus, more therapeutic options are required. Our clinical observations have found that the oral administration of codeine phosphate ameliorated wearing-off in two cases of PD. Case 1 was a woman in her 60s. One day, she had a common cold and took an antitussive, expectorant drug, Aneton, an over-the-counter medicine. After taking it, not only were her common cold symptoms ameliorated but her akinesia also improved. Among the ingredients of Aneton, codeine phosphate proved to be effective for her akinesia. Case 2 was a man in his 50s with PD accompanied by severe, intermittent pain. Codeine phosphate prescribed for his pain improved both his pain and bradykinesia. The oral administration of codeine phosphate ameliorated akinesia/bradykinesia in both cases of PD. This suggests the possible efficacy of codeine phosphate against motor symptoms in PD. In the future, a well-designed multicenter trial is needed to assess the use of codeine phosphate for PD patients, particularly to evaluate its efficacy for motor symptoms, its adverse effects, and its long-term prognosis in patients with PD.

## Introduction

Parkinson’s disease (PD) is the second most common neurodegenerative disease after Alzheimer’s disease. The etiology of PD remains unknown. Neuropathologically, its main lesion is observed in the substantia nigra of the midbrain, where nigral dopaminergic neurons progressively degenerate and disappear. Since the nigral dopaminergic neurons project their axons to the striatum, the concentration of dopamine in the striatum progressively decreases in PD. This decrease in dopaminergic function in the striatum, and its secondary increase in cholinergic activity, causes the main symptoms of PD. Therefore, the mainstay of treatment for PD is to normalize the dopaminergic function in the striatum by administering levodopa, a precursor of dopamine.

PD patients experience a range of motor and nonmotor symptoms. Motor symptoms are well-known and include tremor, rigidity, akinesia/bradykinesia, and postural instability, whereas non-motor symptoms can be diverse and include constipation, olfactory disturbance, neurogenic bladder, orthostatic hypotension, sleep disorders, psychiatric symptoms, cognitive impairment, and pain [1]. The frequency of pain in PD has been reported to be 60.9% (653/1072) [1] and 67.3% (303/450) [2]. Although many drugs for PD with different modes of action, such as levodopa, dopamine agonists, anticholinergic agents, monoamine oxidase B (MAO-B) inhibitors, catechol-O-methyltransferase (COMT) inhibitors, amantadine, zonisamide, etc, are employed in clinical settings, the wearing-off phenomenon and/or non-motor symptoms occur frequently in the advanced stages of the disease; thus, more therapeutic options are required. In this report, we describe two cases of PD, in which the oral administration of codeine phosphate ameliorated their wearing-off phenomenon.

## Case presentation

**Figure 1:**
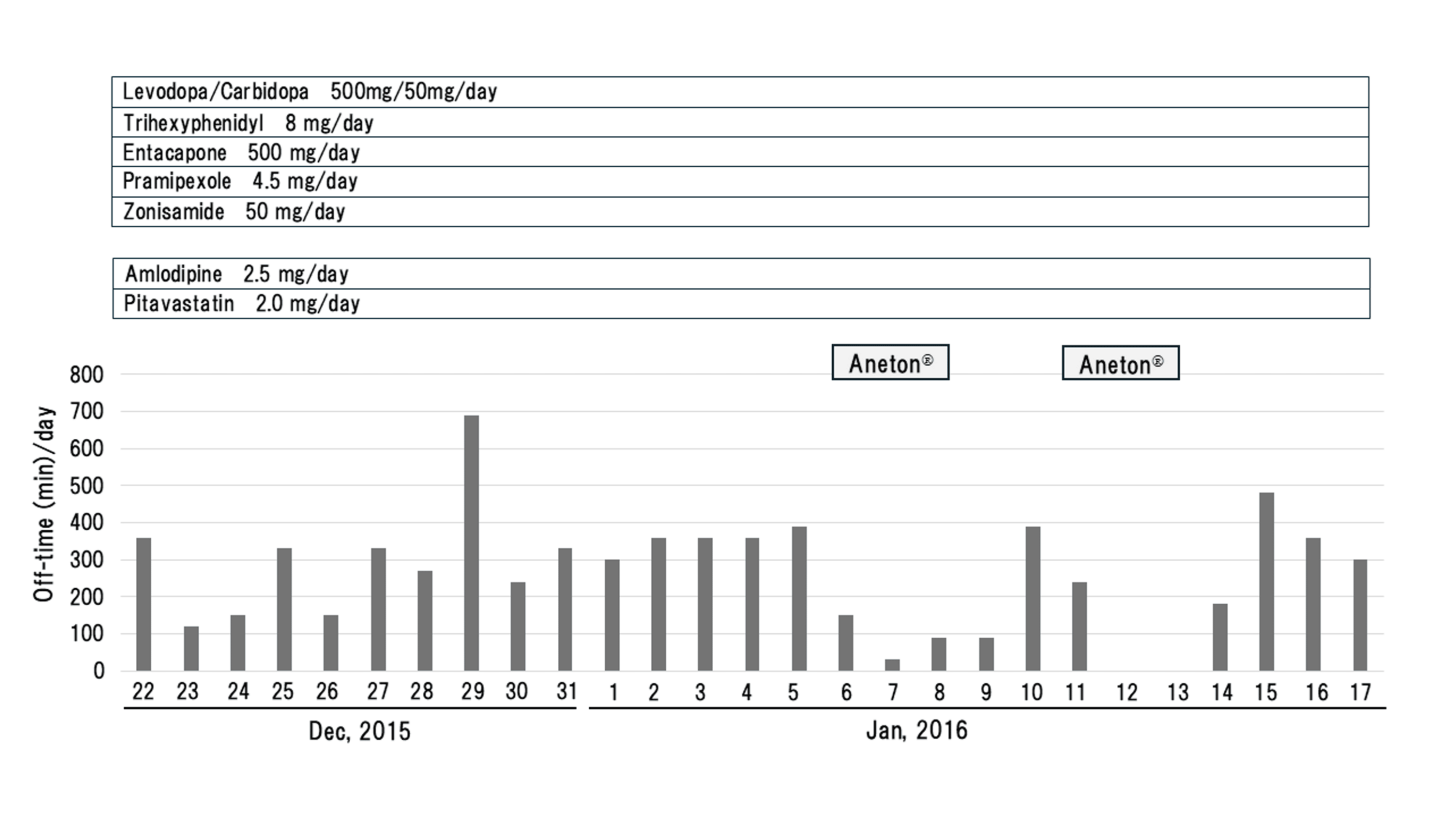
Daily off-time (min) (akinesia) in Case 1. A reduction in daily off-time is observed with Aneton.

Case 1 involved a woman in her 60s, a pharmacist, with a family history of PD; specifically, her father and uncle also had PD. Since her mid-40s, she had shown a slowly progressive deterioration of hand movement and gait, which was diagnosed as PD. Anti-Parkinsonian drugs were prescribed, leading to an improvement in her symptoms. However, as the disease progressed, she required a total of five different types of anti-Parkinsonian drugs (Figure 1).

**Figure 2:**
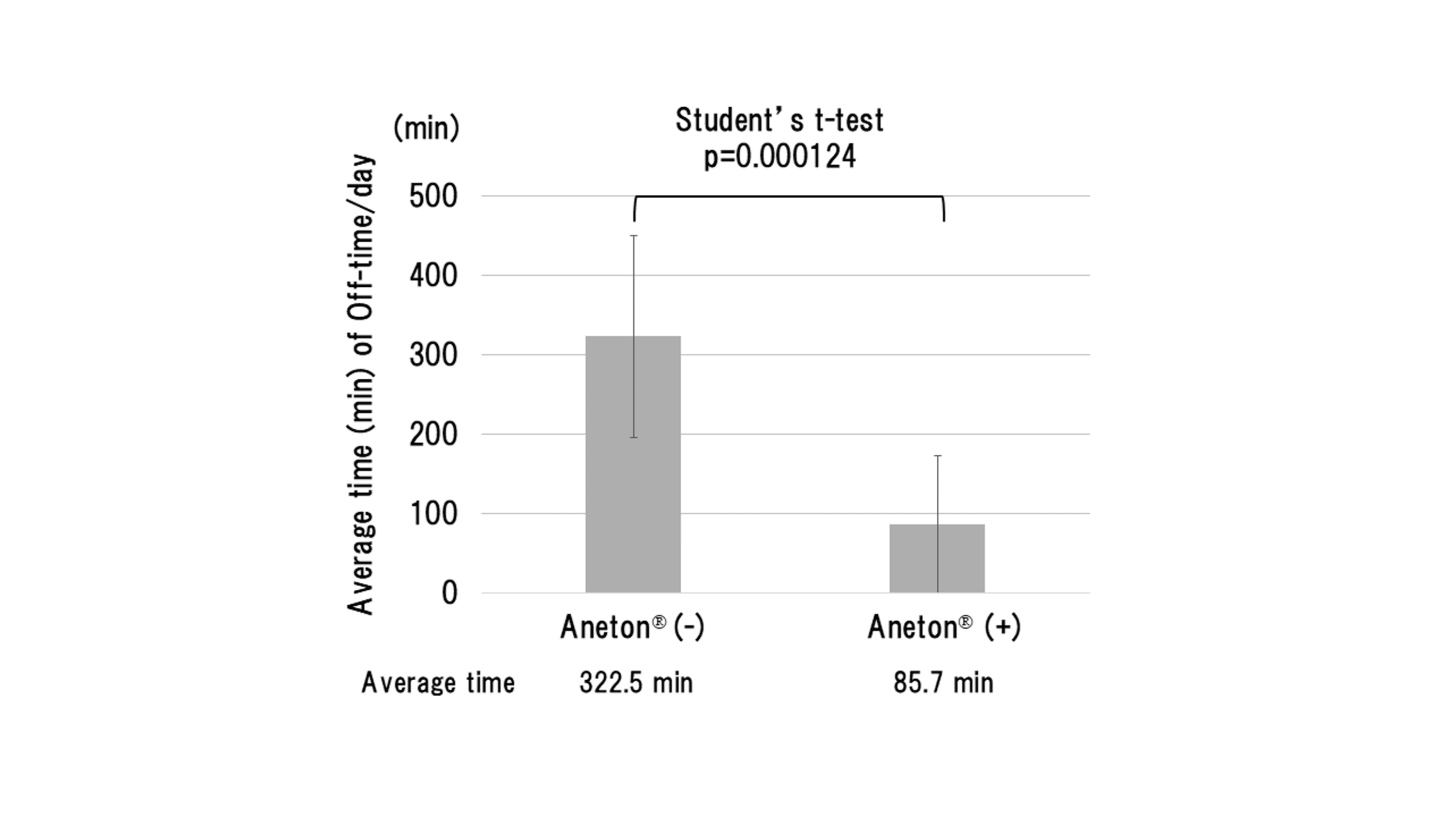
The average time (± SD) of daily off-time (akinesia) in Case 1. The oral administration of Aneton made a statistically significant reduction in average time (min) of daily off-time (p=0.000124 by Student’s t-test). SD: standard deviation

One day, she had a common cold and took an antitussive, expectorant drug, Aneton, an over-the-counter medicine. After taking the medication (3 x 3 tablets/day), not only were her common cold symptoms ameliorated but her akinesia also improved (Figure 1). However, her akinesia worsened again after discontinuation. Therefore, she decided to take Aneton again, and the phenomenon was replicated (Figure 1). She documented her daily "akinesia" (off-time), which was defined as the "inability to walk or perform voluntary movements", half-hourly throughout the daytime (5:30-23:30) in her PD home diary [3]. The average time (± standard deviation) of her daily off-time (akinesia) was 85.7 min (±87.3) and 322.5 min (±127.2) with and without oral administration of Aneton, respectively, which showed a statistically significant reduction induced by Aneton (p=0.000124 by Student’s t-test using EZR software [4]: p<0.05 was considered statistically significant) (Figure 2).

Aneton contains five ingredients, including codeine phosphate, methylephedrine, chlorpheniramine, caffeine, and senega [5]. She asked her doctor to prescribe each ingredient individually, and codeine phosphate proved to be effective for her akinesia. The oral administration of codeine phosphate showed that a reduction in "off time" was observed for an average of four hours per day.

**Figure 3:**
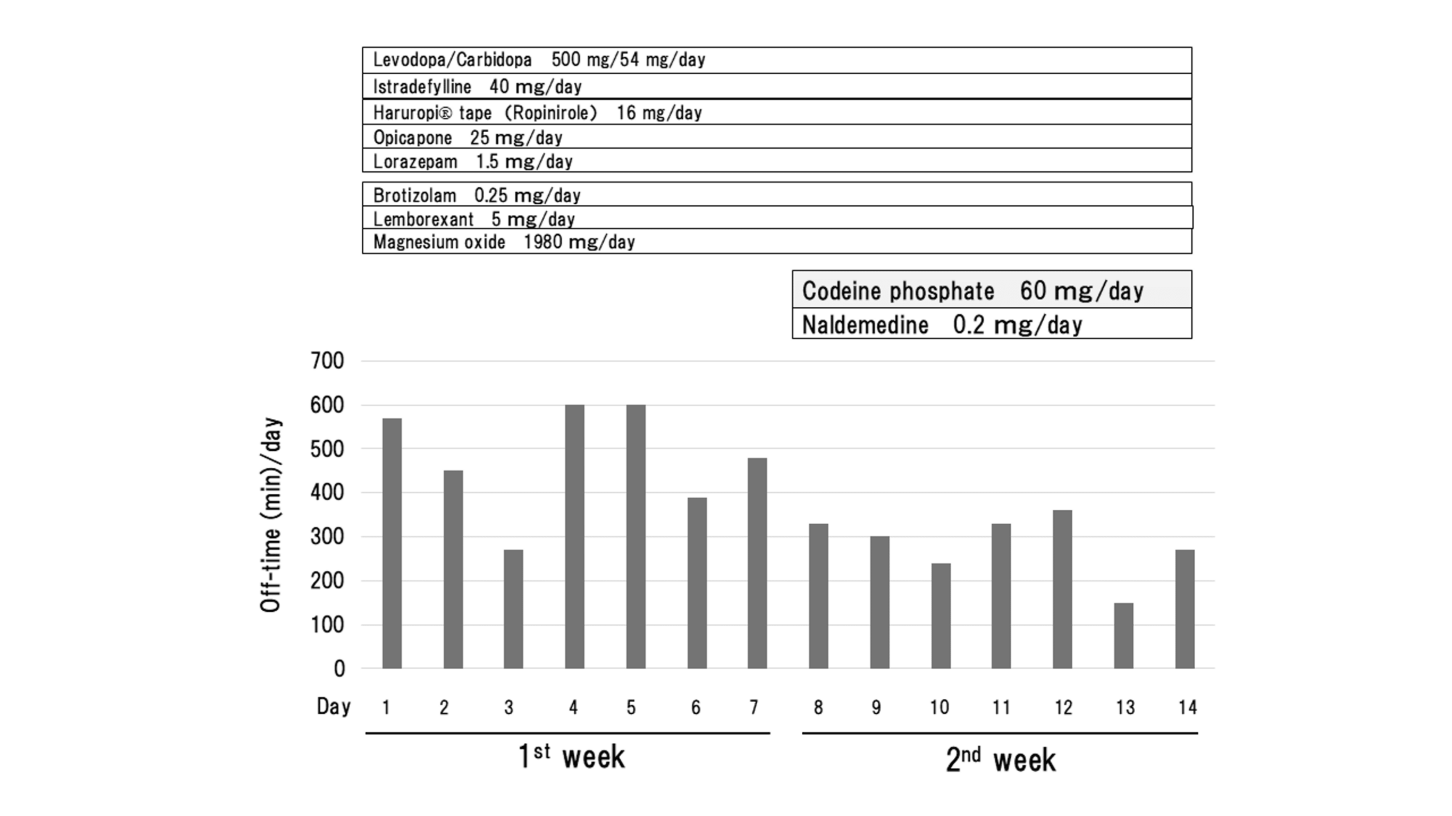
Daily off-time (min) (bradykinesia) in Case 2. A reduction in daily off-time (min) is observed with codeine phosphate.

Case 2 involved a man in his late 50s with PD accompanied by severe, intermittent pain in his right thigh, bilateral buttocks, and lower back. In his early 50s, he started to experience a resting tremor of his right hand, the first symptom of PD. As the disease progressed, rigidity in his extremities, bradykinesia, and slow gait occurred gradually. Brain MRI revealed no abnormalities; however, both dopamine transporter SPECT imaging and ^123^I-MIBG myocardial scintigraphy showed a low uptake of radioisotopes in the striatum and heart, respectively. Because of his severe, intolerable pain, he was once taken to an acute care hospital by ambulance. Extensive investigations including MRI and blood tests were performed, and no abnormal findings were found; therefore, his pain was diagnosed as PD-associated pain. To relieve his pain, he was referred to our hospital and admitted for two weeks. In the first week, he continued to receive his previous prescriptions to observe and evaluate his condition (Figure 3).

**Figure 4:**
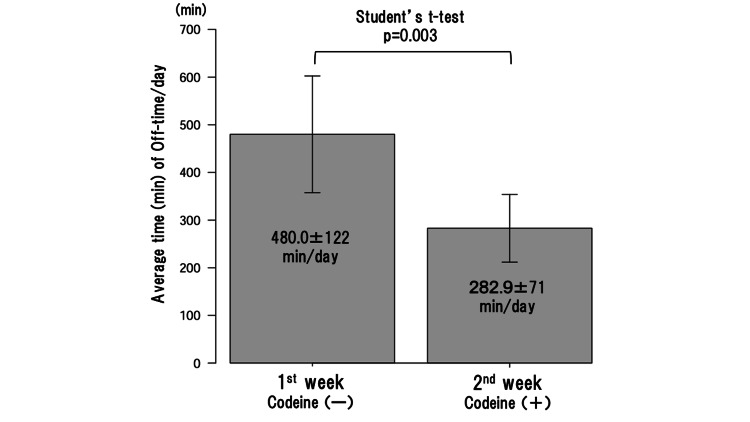
The average time (min) (± SD) of daily off-time (bradykinesia) in Case 2. The oral administration of codeine phosphate made a statistically significant reduction in average time (min) of daily off-time (bradykinesia) (p=0.003 by Student’s t-test). SD: standard deviation

In the second week, after obtaining informed consent, 1% codeine phosphate (3 x 20 mg/day) was prescribed for his pain in addition to the prescribed medications (Figure 3). Naldemedine was added to prevent constipation, a common side effect of codeine. During his stay in the hospital, he could walk and perform movements without assistance; however, during the off-time periods, slow gait and bradykinesia, predominantly on the right side, were observed. The oral administration of codeine phosphate ameliorated both his pain and bradykinesia (Figure 3). The average time (± standard deviation) of his daily off-time (bradykinesia) was 480.0 min (±122) in the first week and 282.9 min (±71) in the second week, showing a statistically significant reduction achieved with codeine phosphate (p=0.003 by Student’s t-test using EZR software [4]) (Figure 4).

**Figure 5:**
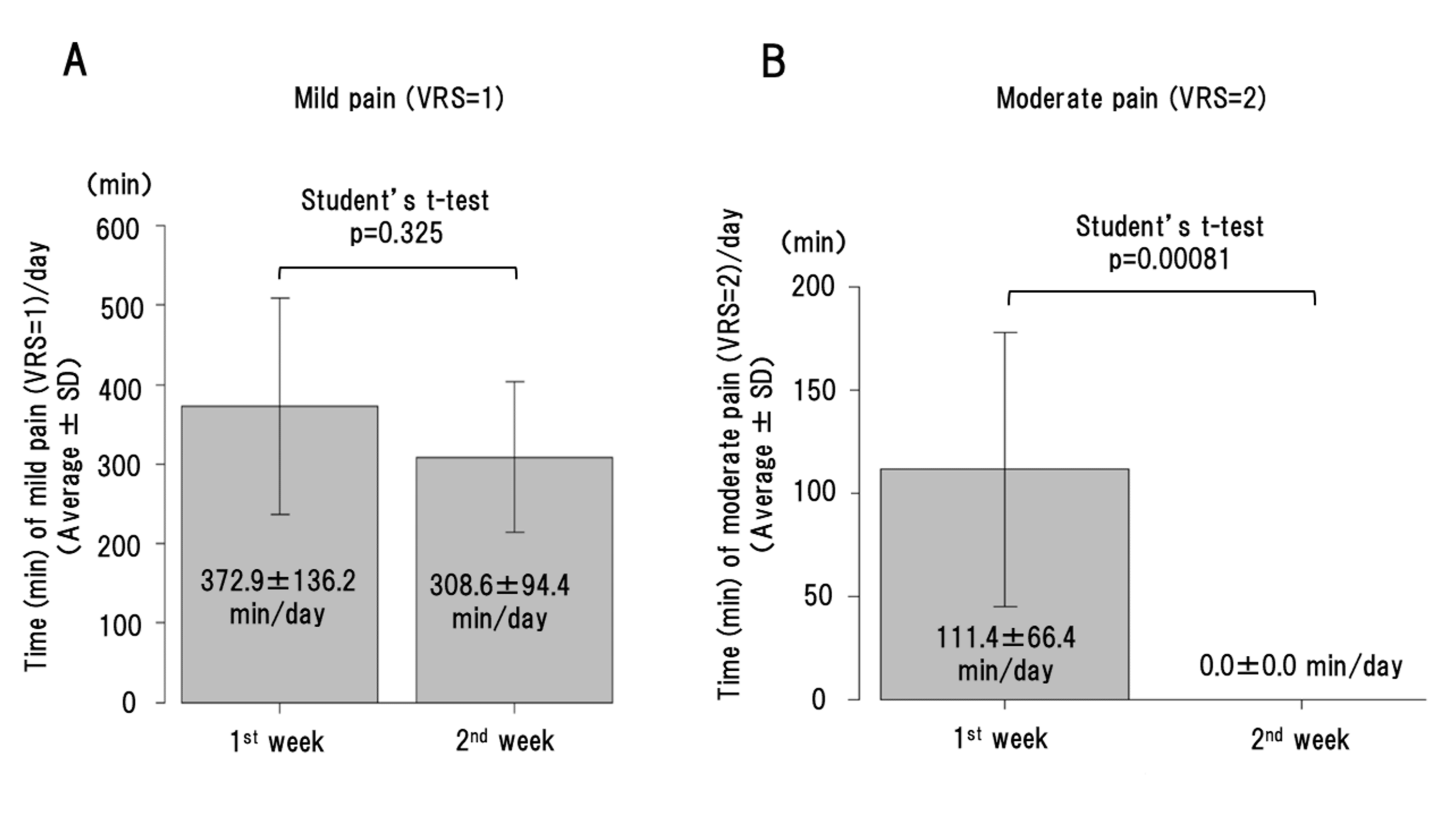
Average time (min) of daily mild pain (A) and moderate pain (B) in Case 2. A: There is no significant difference (p=0.325) in average time (min) of daily mild pain (VRS=1) between without (first week) and with (second week) codeine phosphate. B: Although the average time of daily moderate pain (VRS=2) is 111.4 min in the first week without codeine phosphate, there is no moderate pain in the second week with it. SD: standard deviation; VRS: Verbal Rating Scale

Regarding his pain, it was evaluated by himself using a Verbal Rating Scale (VRS: 0=no pain, 1=mild pain, 2=moderate pain, 3=severe pain). There was no significant difference in average time (min) of daily mild pain (VRS=1) between the first (without codeine phosphate) and second (with codeine phosphate) weeks (Figure 5A).

However, a remarkable difference was observed in average time of daily moderate pain (VRS=2) between the first and second weeks (Figure 5B). Although the patient had a daily VRS=2 pain for 111 min in average in the first week, no pain with VRS=2 appeared in the second week (100% reduction in moderate pain) (Figure 5B). No severe pain (VRS=3) appeared during the hospitalization.

## Discussion

As described above, the patient of Case 1, a pharmacist, asked her doctor to prescribe each ingredient of Aneton individually, and codeine phosphate proved to be effective for her akinesia. The trial of codeine phosphate in Case 1 was conducted in 2015-2016 (Figure 1) before the enforcement of the Clinical Trials Act, Japan, which was established in 2017 and put into effect in 2018. This act regulates clinical trials, including off-label use of pharmaceutical drugs [6]. In Case 2, codeine phosphate was prescribed for his severe pain, and it improved not only his pain but also bradykinesia. 

In both cases of PD, the oral administration of codeine phosphate made a statistically significant reduction in the off-time periods. It is possible that the recovery of common cold symptoms by Aneton in Case 1 and the relief of pain by codeine phosphate in Case 2 may have caused the amelioration of their akinesia/bradykinesia. However, this possibility seems unlikely since the akinesia was ameliorated in Case 1 when Aneton was taken again after the recovery from the common cold and also when codeine phosphate was administered alone as a trial. Currently, the exact mechanism of action by which codeine phosphate improves symptoms remains unknown. Di Chiara and Imperato have reported that the subcutaneous injection of morphine in rats increased the synaptic concentration of dopamine not only in the nucleus accumbens but also in the striatum [7]. Codeine, also known as methylmorphine or methylated morphine, is synthesized from morphine via O-methylation, and both codeine and morphine bind to the μ-opioid receptor [8]. It has also been shown that a fraction of orally administered codeine is converted to morphine in the body [9,10]. Therefore, it seems that the oral administration of codeine phosphate may have increased the concentration of dopamine in the striatum, resulting in the amelioration of akinesia/bradykinesia in both cases.

Codeine is an opioid that is clinically used to manage pain in various diseases, including cancers, and sometimes PD if associated with pain. Edinoff et al. reported that opioids and opioid-like medications such as oxycodone, morphine, tramadol, and codeine are commonly used in the management of chronic pain in PD [11]. Recently, to identify new drug repurposing (repositioning) candidates for PD among existing prescription drugs, Tuominen et al. conducted a nationwide, observational cohort study using Norwegian health registries and found 23 existing drugs, including combined codeine/paracetamol, which were statistically significantly associated with a lower risk of death in patients with PD, suggesting a potential disease-modifying effect [12]. However, it remains unknown whether the effect is due to codeine alone, paracetamol alone, or their combination. The present observations suggest a beneficial effect of codeine for motor symptoms in the two cases of PD. However, PD is a chronic disease and requires long-term use of drugs; so, there is a concern about taking opioids including codeine for a long period of time because of several possible side effects such as nausea/vomiting, constipation, sleepiness, dizziness/vertigo, respiratory insufficiency, confusion, addiction, etc. It is also possible that the anticholinergic side effect of codeine may be enhanced in PD patients treated with an anticholinergic agent, such as trihexyphenidyl in Case 1. At present, the use of codeine should be limited as an option for the management of severe pain in patients with PD. In the future, when clinical observations similar to ours are accumulated, a well-designed multicenter trial is needed to assess the use of codeine for patients with PD, particularly to evaluate its efficacy for motor symptoms, its adverse effects, and its long-term prognosis in patients with PD.

## Conclusions

The oral administration of codeine phosphate made a statistically significant reduction in daily off-time in two patients with PD, suggesting the possible efficacy of codeine phosphate against motor symptoms in PD. These patients are currently being monitored for changes in motor symptoms and their long-term prognosis.
